# Evaluation of early graft function in a case series of lung-transplanted patients

**DOI:** 10.1186/cc13442

**Published:** 2014-03-17

**Authors:** J Fumagalli, I Algieri, M Brioni, AM Villa, GM Ruggeri, F Rapido, A Colombo, S Luoni, G Babini, B Safaee Fakhr, L Spada, S Froio, S Coppola, A Palleschi, L Rosso, D Chiumello, F Valenza, L Gattinoni

**Affiliations:** 1 Fondazione IRCCS Ca Granda - Ospedale Maggiore Policlinico, Milan, Italy

## Introduction

The aim of the study was to investigate early graft function in terms of biological and radiological variables in a case series of lung transplant (Ltx).

## Methods

We performed a prospective analysis of patients that underwent Ltx at Fondazione IRCCS Ca Granda Policlinico of Milan from 1 December 2012 to 10 December 2013. Donors' lung parameters were recorded. Recipients' clinical data were collected daily and lung computed tomography (CT) at end expiration was performed within 7 days after Ltx. On the same day, bronchoalveolar lavage was collected [1]. Quantitative CT analysis was run on separate lungs for each patient.

**Table 1:** Quantitative CT scan analysis on transplanted lungs

Lung CT	Mean ± SD (*n *= 16)
Volume (ml)	1,639 ± 437
Weight (g)	761 ±175
Hyper inflated (%)	0.0 ± 0.0
Normal (%)	38.9 ± 17.1
Poorly (%)	29.4 ± 8.4
Not inflated (%)	31.7 ± 14.9

## Results

Out of 25 LTx, 12 paired data for donors and recipients were analyzed. Lung donors' PaO_2_/FiO_2 _was 392 ± 118, Oto score was 5 ± 3, and ICU length of stay was 2 ± 1 days. Recipients' age was 56 ± 11 years, and body mass index was 24 ± 4 kg/m^2^. Four patients received double Ltx, and the warm ischemia time was 85 ± 16 and 94 ± 14 respectively for right lung and left lung. When the CT scan was performed (on day 4 ± 1), four patients were mechanically ventilated, PaO_2_/FiO_2 _was 270 ± 93 and PaCO_2 _was 44.6 ± 10.0 mmHg. Primary graft dysfunction grade 2 or 3 at 72 hours post Ltx was diagnosed in six patients. All patients were discharged from ICU. Table 1 presents quantitative analysis of transplanted lungs. The alveolar protein concentration and not aerated tissue weight are significantly related (*R*^2 ^= 0.69; *P *= 0.001).

## Conclusion

Alveolar protein concentration is related to morphological features of lungs early after Ltx.
